# Bone Marrow and Wharton’s Jelly Mesenchymal Stromal Cells are Ineffective for Myocardial Repair in an Immunodeficient Rat Model of Chronic Ischemic Cardiomyopathy

**DOI:** 10.1007/s12015-023-10590-6

**Published:** 2023-07-28

**Authors:** Xian-Liang Tang, Marjan Nasr, Shirong Zheng, Taylor Zoubul, Jonah K. Stephan, Shizuka Uchida, Richa Singhal, Aisha Khan, Anna Gumpert, Roberto Bolli, Marcin Wysoczynski

**Affiliations:** 1 Institute of Molecular Cardiology, University of Louisville School of Medicine, Louisville, KY USA; 2 Center for Cardiometabolic Science, University of Louisville School of Medicine, 580 South Preston St. – Rm 204B, Louisville, KY 40202 USA; 3 Center for RNA Medicine, Department of Clinical Medicine, Aalborg University, Copenhagen, Denmark; 4 Interdisciplinary Stem Cell Institute, University of Miami Miller School of Medicine, Miami, FL USA

**Keywords:** Cell therapy, Ischemic cardiomyopaty, Mesenchymal stromal cells

## Abstract

**Background:**

Although cell therapy provides benefits for outcomes of heart failure, the most optimal cell type to be used clinically remains unknown. Most of the cell products used for therapy in humans require *in vitro* expansion to obtain a suitable number of cells for treatment; however, the clinical background of the donor and limited starting material may result in the impaired proliferative and reparative capacity of the cells expanded *in vitro*. Wharton’s jelly mesenchymal cells (WJ MSCs) provide a multitude of advantages over adult tissue-derived cell products for therapy. These include large starting tissue material, superior proliferative capacity, and disease-free donors. Thus, WJ MSC if effective would be the most optimal cell source for clinical use.

**Objectives:**

This study evaluated the therapeutic efficacy of Wharton’s jelly (WJ) and bone marrow (BM) mesenchymal stromal cells (MSCs) in chronic ischemic cardiomyopathy in rats.

**Methods:**

Human WJ MSCs and BM MSCs were expanded *in vitro*, characterized, and evaluated for therapeutic efficacy in a immunodeficient rat model of ischemic cardiomyopathy. Cardiac function was evaluated with hemodynamics and echocardiography. The extent of cardiac fibrosis, hypertrophy, and inflammation was assessed with histological analysis.

**Results:**

*In vitro* analysis revealed that WJ MSCs and BM MSCs are morphologically and immunophenotypically indistinguishable. Nevertheless, the functional analysis showed that WJ MSCs have a superior proliferative capacity, less senescent phenotype, and distinct transcriptomic profile compared to BM MSC. WJ MSCs and BM MSC injected in rat hearts chronically after MI produced a small, but not significant improvement in heart structure and function. Histological analysis showed no difference in the scar size, collagen content, cardiomyocyte cross-sectional area, and immune cell count.

**Conclusions:**

Human WJ and BM MSC have a small but not significant effect on cardiac structure and function when injected intramyocardially in immunodeficient rats chronically after MI.

**Graphical Abstract:**

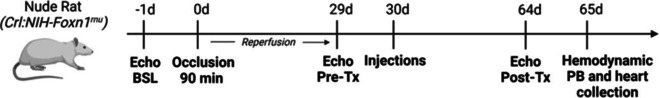

**Supplementary Information:**

The online version contains supplementary material available at 10.1007/s12015-023-10590-6.

## Introduction

Cell therapy has been proposed as a strategy for management of ischemic heart failure in humans [1–3]. Over the past two decades, studies in animal models and in human clinical trials have shown that cell-based reparative approaches for chronic ischemic heart failure are safe and provide benefit [1–4]. Although injected cells do not engraft durably and do not differentiate into cardiomyocytes, they contribute to preservation of ventricular structure and function. To date, several cell types have been used in both pre-clinical models in animals and clinical trials in humans [3, 5–12]. Nevertheless, it remains unresolved what cell type would be the optimal cell product to use in the clinical setting.

Although the heart would seem to be the most logical source of cells for cell therapy, there are some limitations. The most obvious include the donor’s age and comorbidities, and the quality of tissue collected from a diseased heart. The isolation of cells from small endomyocardial biopsies or right atrial appendages collected during bypass surgery require extensive *in vitro* expansion to generate enough cells for treatment [1, 9]. During that time, isolated cells undergo replicative senescence, which limits their proliferative potential and impairs their reparative potential. Collection of tissues from young, healthy donors would be a potential solution; however, myocardial tissue harvesting is an invasive procedure that is expensive and time-consuming, and carries a potential risk of complications.

Given the limitations of aged, diseased hearts as donor sources, bone marrow (BM) became an attractive source of mesenchymal stromal cells (MSCs) used for therapy [1]. Indeed, BM MSCs are a desirable source for organ repair due to accessibility for harvest, propensity to expand in culture to a large number, and biological properties promoting tissue repair. Moreover, their limited immunogenicity after administration in patients makes them suitable as an off-the-shelf allogeneic cell-based product [3].

Umbilical cord tissue (Wharton’s jelly; WJ) contains MSCs similar in morphology and properties to those isolated from BM [13–21]. If effective, WJ MSCs would have obvious advantages as a cell product. The collected tissue is biological waste, so there is no need for potential healthy donor recruitment. Also, because the umbilical cord is new tissue, it contains MSCs with less senescent phenotypes compared to MSCs from other adult tissues used for therapy [13–21]. To date, there are no studies comparing the myocardial reparative potential of these two cell types. The present study addresses this critical gap in knowledge.

Here, we tested the reparative potential of human WJ MSCs and BM MSCs. We used an immunodeficient rat model of chronic ischemic cardiomyopathy and intramyocardial injection of cells. We assessed cardiac function with hemodynamic studies and echocardiography. We hypothesized that WJ MSCs have superior reparative potential compared with BM MSCs when injected in the chronic phase of MI due to their less senescent phenotype and superior pro-reparative secretome.

## Material and Methods

### Human BM and WJ Stromal Cell Culture

Human BM MSCs and WJ MSCs were a kind gift from Dr. Aisha Khan (University of Miami Interdisciplinary Stem Cell Institute). Manufacturing methods, including cell expansion, cryopreservation, shipment, and release testing, have been described [22]. BM MSCs and WJ MSCs were cultured in MEM medium (Gibco) supplemented with 20% FBS (Seradigm), 0.2 mM L-glutamine (Gibco), and 100 U/ml penicillin/streptomycin (Gibco). BM MSCs and WJ MSCs were not used beyond passage number six. All cell lines were maintained under standard incubation conditions at 37 °C with 5% atmospheric CO_2_ and passaged using TrypLE™ (ThermoFisher Scientific) when approaching ~ 70% confluence.

### Brightfield Imaging

At passage 4–6, cells were detached from culture flasks with 0.25% trypsin–EDTA, washed in PBS, and suspended in a growth media. Subsequently, cells were seeded on glass bottom dishes at 1 × 10^5^ cells per dish. After 48 h, brightfield images were collected with a Keyence BZ-X810 imaging system under 4 × objective and 1.5 × digital zoom.

### Flow cytometry

Cells were detached from culture dishes with 0.25% trypsin–EDTA. After incubation for 30 min at 4 °C with monoclonal antibodies, cells were washed, suspended in 0.5 mL of PBS, and analyzed by flow cytometry with BD LSRFortessa system. Post-acquisition analysis to determine expression of detected markers was performed with FlowJo software.

### Proliferation and Cell Population Doubling Time

Cell proliferation was assessed by counting cells using a BD LSRFortessa flow cytometer. Briefly, 20,000 cells were seeded per well in complete growth medium in 12-well plates. Twenty-four hours later, a group of cells was counted and recorded as time = 0 h. The medium in the remaining plates was then replaced with fresh growth medium, and the cells were cultured for the indicated durations. The cells were harvested by trypsinization and suspended in growth medium. A small volume of suspended cells was used to record cell events using the BD LSRFortessa flow cytometer, with proper gating to exclude cell debris. To calculate the cell doubling time (Td), the following formula was used: Td = Tln2/ln(Xe/Xb), where T is the incubation time, Xb is the cell number at the beginning of the incubation time (i.e., t = 0 h), and Xe is the cell number at the end of the incubation time; Xe and Xb were recorded in the log phase of cell growth.

### EdU Assay

The EdU cell proliferation assay was performed per manufacturer recommendation (ab219801, Abcam). Briefly, 1 × 10^5^ cells were seeded on 35-mm glass bottom tissue culture dishes (MatTek Corporation) in growth media. At ~ 70–80% confluence, cells were incubated for 6 h with 5-ethyl-2-deoxyuridine (EdU, 10 µM), which incorporates into the newly synthesized DNA. After fixation, permeabilization, and blocking of the cells, EdU incorporation was visualized using the Click it EdU-Alexa-Flour488 imaging kit. The cells were treated with DAPI to visualize nuclei (Thermo Fisher).

### Immunocytochemistry

Briefly, 1 × 10^5^ cells were seeded on 35-mm glass bottom tissue culture dishes (MatTek Corporation) in growth media. At ~ 70–80% confluence, cells were fixed, permeabilized with freeze cold methanol for 10 min, and washed in PBS. Subsequently, cells were labeled with primary antibodies: anti-phospho-Histone H3 (Ser10) (6G3) (Cell Signaling, #9706) anti-Ki-67 (8D5) (#9449, Cell Signaling). After overnight staining at 4˚C, cells were washed in PBS and stained with secondary antibody (Anti-mouse IgG (H + L), F(ab')2 Fragment [Alexa Fluor® 488 Conjugate] #4408 Cell Signaling) at room temperature. After 2 h, the cells were washed in PBS and stained with Hoechst H33342 (5 µg/mL) to visualize nuclei. Stained cells were imaged within 24 h with the Keyence BZ-X810 imaging system.

### Beta-Gal Activity

WJ and BM MSCs 1 × 10^5^ were seeded on 35 mm glass bottom tissue culture dishes (MatTek Corporation) in growth media. At ~ 70–80% confluence, cells were fixed and Beta-Gal activity was detected with a kit per manufacturer’s recommendation. Stained cells were imaged with brightfield microscopy with the Keyence BZ-X810 imaging system.

### RNA Isolation and Quantification

WJ and BM MSC RNA was extracted with TRIzol per manufacturer’s indications. Extracted RNA was quantified using a NanoDrop 2000C. RNA with OD260/280 > 2.0 was used for RNA-seq.

### RNA-seq and Bioinformatic Analysis

RNA samples were shipped to Novogene to perform RNAseq analysis. Quality control (QC) of the raw sequence data was performed using FastQC (version 0.10.1) for each sequencing sample. The sequences were aligned to the *Hs* reference genome assembly (GRCh38.93) using STAR version 2.6 [23]. Differential expression of ENSEMBL protein-coding transcripts was performed using DESeq2 [24]. For the analysis using DESeq2, the raw counts were obtained from the STAR-aligned bam format files using HTSeq version 0.10.0 [25]. The raw counts were normalized using the Relative Log Expression (RLE) method and then filtered to exclude genes with fewer than ten counts across the samples. DESeq2 guidelines were used to identify differentially expressed genes, and all p-values were adjusted for testing multiple genes (Benjamini–Hochberg procedure; *p* ≤ 0.05). Functional enrichment analysis was performed using the clusterProfiler R package to identify enriched Gene Ontology biological processes and KEGG pathways for each set of differentially expressed genes (DEGs) [26].

### Ischemia–Reperfusion; Cell and Vehicle Administration

All animal experiments were performed in accordance with the Guide for the Care and Use of Laboratory Animals published by the U.S. National Institutes of health (revised 2020) and with the guidelines of the Animal Care and Use Committee of the University of Louisville, School of Medicine (Louisville, KY, USA), and therefore were carried out in accordance with the ethical standards laid down in the 1964 Declaration of Helsinki and its later amendments. Similar to our previously performed ischemia–reperfusion injury studies [10], female athymic homozygous nude rats (genotype, *Foxn1*^*rnu/rnu*^; supplier, ENVIGO Laboratories; age 2–3 months; weight 150–180 g) were anesthetized with ketamine (37 mg/kg) and xylazine (5 mg/kg) and intubated with a rodent respirator (Harvard Apparatus). Anesthesia was maintained with isoflurane inhalation and body temperature sustained at 37 °C with a heating pad. Following administration of antibiotics, the chest was opened via left thoracotomy and the heart exposed. All animals underwent a 90-min occlusion of the left anterior descending coronary artery followed by reperfusion, after which the chest wall was sutured closed. Thirty days after reperfusion, rats were re-anesthetized, and the heart exposed via a left anterolateral thoracotomy. At this time, rats received four intramyocardial injections of BM MSCs, WJ MSCs, or vehicle (1 × PBS; phosphate-buffered saline) in the peri-infarct region. In effort to limit donor-dependent variables (e.g., disease state, age, gender, etc.), each animal received a mixture of BM MSCs and WJ MSCs sourced from three randomly selected donors. The BM MSCs and WJ MSCs groups received a total of 3 million cells per heart (four injections; 750,000 cells per injection). All groups were euthanized 35 days after injection (65 days after MI).

### Echocardiographic Procedures and Analyses

**Fig. 1:**
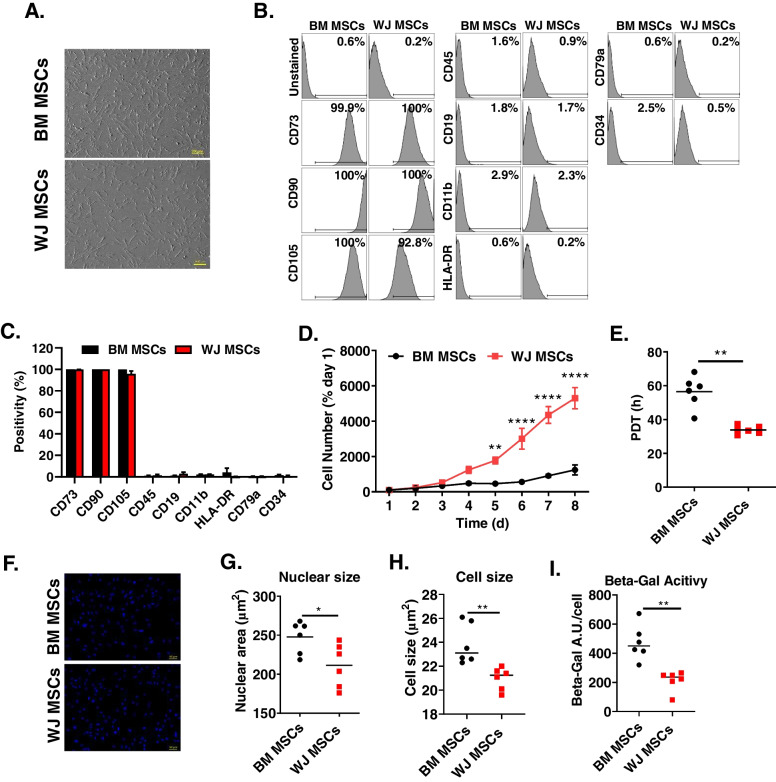
BM and WJ MSC morphology, immunophenotype, and proliferative capacity. **(A)** BM and WJ MSCs were seeded on glass bottom dishes in growth media. After 48 h of culture cell were imaged with brightfield microscope. **(B)** BM MSCs and WJ MSCs were labeled with monoclonal antibodies and analyzed with flow cytometry. Represtantive histograms are shown. **(C)** Numerical representation of the flow cytometric data. The data are mean ± SEM, *n* = 3. **(D)** Proliferation curves of synchronized cultures of BM MSCs and WJ MSCs. **(E)** Population doubling time (PDT) of BM MSC and WJ MSC synchronized cultures. **(F)** Immunofluorescence images of BM MSCs and WJ MSCs labeled with Hoechst 33342 and **(G)** quantification of nuclear area. **(H)** Analysis of BM MSCs and WJ MSCs cell size of detached cultures. **(I)** Comparison of BM MSCs and WJ MSCs beta-galactosidase activity. E, G, H, and I show individual data points, horizontal lines represent mean values. BM MSCs *n* = 6, WJ MSCs *n* = 6; *P < 0.05, **P < 0.01 (unpaired t test)

Indices of cardiac function and ventricular dimensions were assessed via echocardiography under light anesthesia (pentobarbital, 25 mg/kg, i.p.), as previously described [10] with serial measurements obtained at baseline (prior to MI), 29 days post-MI (prior to treatment), and 64 days post-MI (34 d after treatment) (Fig. 1). The anterior chest was shaved, and rats were placed in the left lateral decubitus position. Body temperature was maintained between 36.9 °C and 37.3 °C. Echocardiographic images were acquired using a Vevo 2100 Imaging System equipped with a 20-MHz transducer. Hearts were imaged in the para-sternal long axis view to measure LV end-systolic and end-diastolic volumes (LVESV and LVEDV, respectively), as well as ejection fraction (EF). All measurements were averaged in three consecutive cardiac cycles and analyzed off-line by a single blinded observer. All calculations were derived using standard formulas and analyzed according to modified American Society for Echocardiography standards. Echocardiograms were collected from all 54 animals.

### Hemodynamic Procedures And Analyses

Hemodynamic studies were performed 65 days after MI, just prior to euthanasia as previously described [10]. Rats were anesthetized with ketamine (37 mg/kg) and xylazine (5 mg/kg), intubated, and mechanically ventilated. Anesthesia was maintained with 1% isoflurane and the core temperature sustained at 37 °C with a heating pad for the duration of the procedure. A 2F micro-tip pressure–volume (PV) catheter (SPR-869, Millar Instruments) was inserted into the right carotid artery and advanced into the LV cavity. The right jugular vein was cannulated for fluid administration. After 20 min of stabilization, the PV signals were recorded continuously with an ARIA PV conductance system (Millar Instruments) coupled with a Powerlab/4SP A/D converter (AD instruments), stored, and displayed on a personal computer. Pressure–volume relationships were assessed by transiently compressing the inferior vena cava with a cotton swab. Parallel conductance from surrounding structures was calculated by injecting a small bolus of 15% NaCl through the jugular vein. LV end-diastolic pressure (LVEDP), dP/dt_max_ and dP/dt_min_, end-systolic elastance (Ees), and adjusted maximal power were calculated using the PVAN software program Millar [9]. Conductance catheterization was performed on all 54 animals. One animal was excluded from the final analysis due to poor quality of readings.

### General Histology and Morphometry Procedures

Following hemodynamic measurements, a polyethylene catheter filled with phosphate buffer (0.2 M, pH 7.4) and heparin (100 IU/ml) was advanced to the ascending aorta via the right carotid artery. In rapid succession, the heart was arrested in diastole by injecting 1 ml of a mixture of cadmium chloride (100 mM) and potassium chloride (3 M) through the aortic catheter. Subsequently, the heart was excised and retrogradely perfused with phosphate buffer for 3 min to clear residual blood within the coronary circulation, and perfused with 10% neutral buffered formalin solution for 15 min. Perfusion pressures were maintained between 60 and 80 mmHg while end-diastolic pressures were kept at 8 mmHg. After perfusion-fixation, the atria and right ventricle were dissected from the left ventricle. The LV weight was measured and recorded. Each heart was then cut into five transverse slices (each 3 mm thick), processed, paraffin-embedded, and sectioned into 5 µm thick sections.

### Scar Size Determination by Masson’s Trichrome Staining

Masson’s trichrome staining was performed according to the manufacturer’s instructions (HT15-1KT; Sigma-Aldrich) as previously described [10]. Briefly, formalin-fixed, paraffin-embedded myocardial tissue sections were heated at 80 °C for 30 min, deparaffinized in xylene, and stepwise rehydrated via incubation in decreasing concentrations of ethanol (100, 96, 90, and 80%) and deionized water. Tissue sections were subsequently incubated in Bouin’s solution (Thermo Fisher Scientific) at 55 °C for 15 min and left to cool for 5 min. Sections were then washed with deionized water for 15 min, left to rest in deionized water for 5 min, and then stained with Biebrich Scarlet-Acid Fuchsin solution (HT151, Sigma-Aldrich) for 10 min. Tissue sections were next rinsed in deionized water for 5 min and placed in working phosphotungstic acid (HT152, Sigma-Aldrich)/phosphomolybdic acid (HT153, Sigma-Aldrich)/deionized water solution for 5 min. Sections were successively counter-stained in aniline blue (Sigma-Aldrich) for 10 min, rinsed in 1% acetic acid for 1 min, and stepwise dehydrated with incubation in increasing concentrations of ethanol (80, 90, 96, and 100%). Tissue sections were finally mounted under glass coverslips using Permount Mounting Media (Fisher Scientific). Images were digitally acquired using a BZ-X810 imaging system (Keyence) and analyzed using BZ-X800 software (Keyence). Morphometric parameters, including total LV area, remote area, risk area, and scar area, were measured in each section. In accordance with our previous work [27], the risk region was defined as the sum of the LV segment containing the infarct scar and the two border zones (the regions that encompass 0.5 mm on either side of the lateral borders of the scar). Total scar size (mass in mg) was enumerated by summation of scar masses corresponding to each transverse myocardial slice (apex to base; five slices per heart) [scar mass (mg) = section mass(mg)(scar area(mm^2^)LV area(mm^2^))][scar mass (mg) = section mass(mg)(scar area(mm^2^)LV area(mm^2^))].

### Quantification of Myocardial Collagen Content by Picrosirius Red Staining

To evaluate cardiac fibrosis, myocardial collagen content was enumerated in picrosirius red stained sections via quantitative analysis of polarized light microscopy images, as previously described [10]. As above, formalin-fixed, paraffin-embedded myocardial tissue sections were heated, deparaffinized, and rehydrated. Picrosirius red stain was prepared using 0.1% (w/v) Direct Red 80 (Sigma-Aldrich) in picric acid (Sigma-Aldrich). For each heart, mid-papillary muscle level myocardial sections were incubated in picrosirius red for 1 h, washed in 0.5% acetic acid (2 times; 1 min each), dehydrated with ethanol, and mounted under glass coverslips using Permount Mounting Media (ThermoFisher Scientific). Images were digitally acquired using a BZ-X810 imaging system (Keyence) and analyzed using BZ-X800 software (Keyence). Results are expressed as the percent area of collagen per myocardial tissue region.

### General Immunostaining Procedures

Transverse myocardial tissue sections were heated at 80 °C for 30 min, deparaffinized with xylene, and stepwise rehydrated by successive rinsing in decreasing concentrations of ethanol (100, 96, 90, and 80%) prior to placing in deionized water. Antigen retrieval was then performed by submerging tissue sections in citrate retrieval buffer [2.4 g/L sodium citrate tribasic dehydrate (S4641, Sigma-Aldrich), 0.35 g/L citric acid (C0759, Sigma-Aldrich), pH 6.0] for 10 min at approximately 100 °C. Sections were allowed to cool for 10 min on ice and washed in deionized water prior to application of diluted antibodies. Primary and secondary antibodies were diluted in antibody diluent reagent (003218, Invitrogen) prior to use. All sections were counterstained with 1 µg/mL DAPI (D3571, Invitrogen) for 10 min and subsequently incubated with 0.1% Sudan Black solution (199664, Sigma-Aldrich) in 70% ethanol for 15 min to mitigate the autofluorescence signal. Sections were then washed with 1 × PBS (3 times; 3 min each), rinsed in deionized water, and finally mounted under glass coverslips using PermaFluor Aqueous Mounting Medium (TA-030-FM, ThermoFisher Scientific). Images were acquired digitally using a Nikon Eclipse Ti fluorescence microscope and analyzed using NIH Image J software (1.46r).

### Cardiomyocyte Cross-Sectional Area

To assess cardiomyocyte cross-sectional area, mid papillary heart sections were stained with rhodamine-conjugated WGA (to demarcate cell membranes) for 1 h at 37 °C, and subsequently washed in 1 × PBS. Next, slides were counterstained with DAPI, autofluorescence was blocked with 0.1% Sudan Black solution, and mounted under glass coverslips. Myocardial regions corresponding to peri infarct and remote zones were imaged using the BZ-X810 imaging system (Keyence) and analyzed using BZ-X800 software (Keyence). Cardiomyocyte areas were determined in transversely-cut cells with centrally located nuclei.

### Hemavet Analysis

Peripheral blood was collected at the time of euthanasia from the vena cava into EDTA coated tubes. The sampled were analyzed within 24 h of collection with a Hemavet 950 (Drew Scientific) to obtain complete blood count (CBC) including counts of white blood cells (WBCs), neutrophils, lymphocytes, and monocytes per µL of blood.

### Statistical Analyses

Statistical analyses were performed using GraphPad Prism version 9.00 for Windows (GraphPad Software, La Jolla California USA, http://www.graphpad.com). Echocardiographic and hemodynamic values were subject to 1-way or 2-way ANOVA, as appropriate, followed by the *post hoc* Holm–Sidak’s multiple comparison test. For the cardiomyocyte cross-sectional area data (which did not exhibit a Gaussian distribution), statistical significance was assessed using the non-parametric Kruskal–Wallis test followed by a *post hoc* Dunn’s multiple comparison test. For all data sets, the arithmetic mean ± SEM is reported. *P* values < 0.05 were considered statistically significant.

## Results

### BM MSC and WJ MSC Morphology And Immunophenotype

MSCs are a heterogenous population of mesenchymal stromal cells residing in various tissues to support parenchymal cell function. Thus, we performed assessment of BM and WJ MSC morphology and immunophenotype. We found that both BM MSCs and WJ MSCs adhere to the tissue culture dishes, possess fibroblast-like morphology, and were morphologically indistinguishable (Fig. 1A). Next, we performed flow cytometry to determine WJ and BM MSCs immunophenotypes. We found that both cell types express classical markers of mesenchymal cells, i.e., CD73, CD90, and CD105. They were null for the hematopoietic and endothelial markers CD45, CD19, CD11b, HLA-DR, CD79b, and CD34. The level of expression of these markers was not different between BM MSCs and WJ MSCs (Fig. 1B and C). These data suggest that both cell types are of mesenchymal origin. Next, we tested their proliferative capacity.

### Proliferative Capacity of BM MSCs and WJ MSCs

BM and WJ MSC cell number were monitored in synchronized cell cultures in optimal growth medium. BM MSCs showed a linear increase in cell number as monitored daily; WJ MSCs showed a more robust increase in cell number, reaching full confluence at day 8 (Fig. 1D), which translated into a reduced population doubling time in WJ MSCs compared with BM MSCs (33.9 ± 1.0 vs 56.5 ± 3.8 h) (Fig. 1E). The absolute cell number in the cultures is determined by the rate of proliferation and the rate of cell death. Thus, next we evaluated the rate of proliferation with EdU staining. BM MSCs and WJ MSCs were propagated in optimal growth medium. BM MSCs showed a slower rate of proliferation (9.9 ± 3.5%) compared with WJ MSCs (41.2 ± 3.0%) (Supplementary Fig. 1A). Next, we evaluated the percentage of cells that actively engaged in the cell cycle with Ki67 staining. We found that BM MSCs compared with WJ MSCs have smaller percentages of cells actively cycling, according to Ki67 staining (36.6 ± 8.2 vs 81.9 ± 2.5%) (Supplementary Fig. 1B). Similarly, the percentage of cells engaged in the M-phase evaluated with phospho-histone H3 staining showed smaller proportion in BM MSCs compared with WJ MSCs (0.7 ± 0.1 vs 1.6 ± 0.1%) (Supplementary Fig. 1C). These data suggest that WJ MSCs have higher proliferative capacity than BM MSCs. Because WJ MSCs are isolated from neonatal tissue, their superior proliferative capacity may be related to less cell proliferative senescence. Thus, we next quantified hallmarks of cell senescence *in vitro*.

### Proliferative Senescence Markers in BM MSCs and WJ MSCs

Senescent cells are characterized by increased cell size, which is often accompanied by an increase in nuclear size. To ascertain the magnitude of BM and WJ MSC senescence, cells were labeled with Hoechst 33342 to visualize cell nuclei, imaged, and subjected to analysis to determine nuclear size. The nuclear size in BM MSCs was significantly higher compared with WJ MSCs (247.8 ± 8.4 vs 211.4 ± 11.4 µm^2^) (Fig. 1F and G). Furthermore, brightfield images of detached cells showed that BM MSCs compared with WJ MSCs have larger diameter (23.8 ± 0.7 vs 21.0 ± 0.4 µm) (Fig. 1H). These data suggest that WJ MSCs compared with BM MSCs have a less senescent phenotype. Cell senescence is associated with increased lysosomal β-galactosidase (β-Gal) activity measured at pH 6.0. Next, we subjected cells to β-Gal activity assays. We found that β-Gal activity was higher in BM MSCs compared with WJ MSCs (473.1 ± 49.1 vs 212.1 ± 27.8 AU/cell) (Fig. 1I). Together, these data suggest that WJ MSCs have a less senescent phenotype than BM MSCs.

### Transcriptomic Analysis of BM MSCs and WJ MSCs with RNAseq

**Fig. 2:**
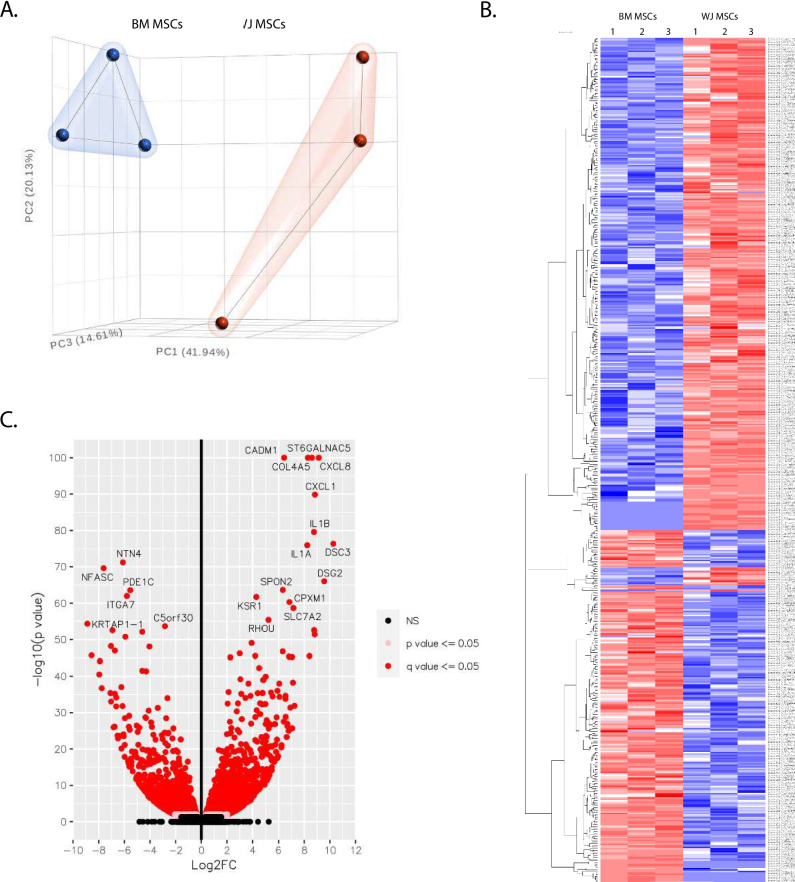
RNAseq analysis of BM MSCs and WJ MSCs. Three dimensional PCA plot performed on normalized read for BM and WJ MSCs samples **(A)**. Heatmap of upregulated and down regulated transcripts in BM MSCs and WJ MSCs **(B)**. Volcano plot of transcripts upregulated and downregulated in BM MSCs and WJ MSCs samples **(C)**

To determine whether the tissue origin impacts MSCs, we performed a transcriptional profile of BM MSCs and WJ MSCs by unbiased RNA-seq analysis. Unsupervised analysis of the RNA-seq with principal component analysis (PCA) shows group separation between BM MSCs and WJ MSCs and indicates that these cells have distinct transcriptional profiles (Fig. 2A). The heat map analysis shows that out of 2517 differentially expressed transcripts, 1417 are upregulated and 1100 are downregulated in WJ MSCs compared with BM MSCs (Fig. 2B). Gene Ontology (GO) enrichment analysis of differentially expressed genes indicates that compared with BM MSCs, WJ MSCs differentially express genes involved in extracellular matrix organization, extracellular structure organization, regulation of MAP kinase activity, regulation of protein serine/threonine kinase activity, response to mechanical stimulus, and glycosaminoglycan biosynthesis, among others (Supplementary Fig. 2A). Kyoto Encyclopedia of Genes and Genomes (KEGG) analysis showed that differentially express genes in WJ MSCs as compared with BM MSCs are involved in TNF, AGE-RAGE C-type lectin, PI3K-Akt, MAPK, TGF-beta, and IL-17 signaling pathways, among others (Supplementary Fig. 2B). Recent observations suggest that cell administration in hearts after MI elicits recruitment of immune cells to promote myocardial repair. The differentially expressed transcripts in WJ MSCs, compared with BM MSCs, were analyzed to examine the distribution of fold difference with respect to significance levels (Fig. 2C). We found that among the most enriched genes in WJ MSCs, compared with BM MSCs, are CXCL8, CXCL1, CXCL3, IL1a, and IL1b, factors that are instrumental for recruitment of immune cells and activation of inflammatory pathways. Next, we measured CC and CXC chemokines in conditioned media from WJ MSCs and BM MSCs. We found that WJ MSCs, compared with BM MSCs, secrete abundant CC and CXC chemokines. These include CCL2, CCL7, CXCL1, CXCL2, CXCL5, CXCL6, and CXCL8. These chemokines are known ligands for CXCR1, CXCR2, and CCR2 receptors, which are involved in recruitment of immune cells (Supplementary Fig. 3A and B). Together, these data suggest that BM MSCs and WJ MSCs have a distinct secretome profile relevant to myocardial repair.

### Rat model of Ischemic Cardiomyopathy

**Fig. 3:**
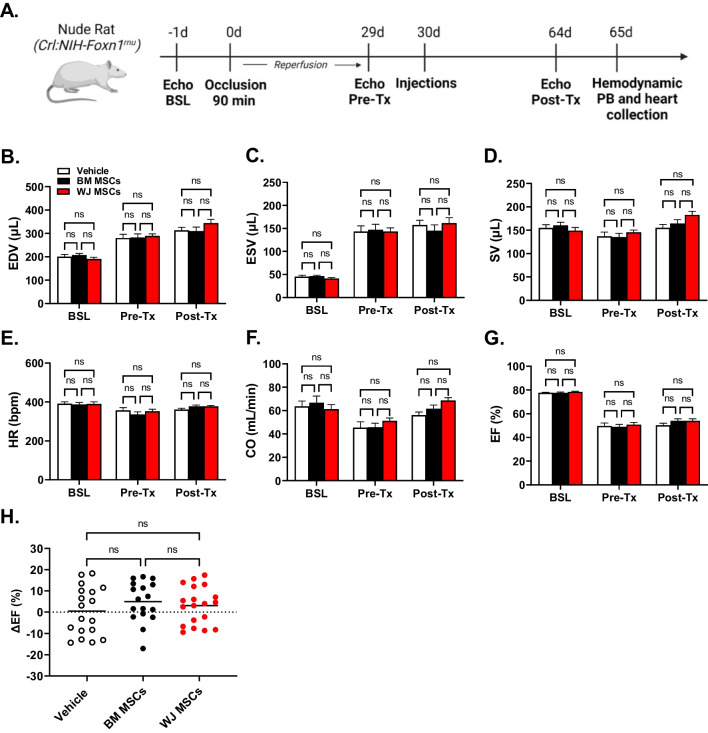
Impact of BM and WJ MSC treatment on ventricular volumes and function measured with echocardiography. **(A)** Female nude rats were subjected to 90 min ischemia. After 30 d of reperfusion rats were injected with vehicle, WJ MSCs, or BM MSCs (3 × 10^6^, intramyocardially). At 65 d rats were subjected to hemodynamic study and euthanized for tissue collection. During the study rat heart function was evaluated with echocardiography at the baseline (Echo BSL), before injections (Echo Pre-Tx), and one day before completion of the study (Echo Post-Tx). Illustration prepared with BioRender. At associated time points, ventricular volumes (**A** end-diastolic volume [EDV] and **B** end-systolic volume [ESV]) and functional parameters (**C** stroke volume [SV], **D** heart rate [HR], **E** cardiac output [CO], **F** ejection fraction (EF)) were calculated for each group. The change in **G** ejection fraction (ΔEF) from pre-treatment to post-treatment is depicted in accompanying dot plot. Graphs report mean ± SEM for treatment groups or individual point with mean consisting of vehicle (*n* = 18), BM WJ MSCs (*n* = 17), and BM MSCs (*n* = 19) injected animals; ns – not significant *(*two-way ANOVA)

To test the reparative potential of human WJ MSCs and BM MSCs, we used an immunodeficient rat model of ischemic cardiomyopathy as we described before [10]. Left ventricular function was evaluated via echocardiographic measurements one day before occlusion (-1 d), before cells or vehicle injections (29 d), and one day before experiment termination (64 d). Furthermore, we assessed LV function with hemodynamic measurements at the end of the study (65 d), before heart collection for histology (Fig. 3A). A total of 76 rats were enrolled in the study and underwent MI. Sixteen rats died after MI before group assignment, and three were euthanized due to infection. The remaining 57 rats were randomly assigned to one of the three groups (vehicle, WJ MSCs, or BM MSCs). Two rats died after treatment and one was euthanized due to infection. Thus, a total of 54 rats (18 vehicle, 17 WJ MSCs, and 19 BM MSCs) completed the study and were included in the final analysis. The exclusion criterion set a priori was an ejection fraction drop greater than 20 points from the baseline at 30 d after occlusion [27, 28]; however, none of the rats showed a reduction in EF < 20 points prior cell or vehicle injection; thus, no additional exclusions were applied.

### Effect of WJ MSCs and BM MSCs on Left Ventricular (LV) Function Measured with Echocardiography

Echocardiographic analysis performed prior MI (BSL, -1 d) and before cell injection (Pre-TX, 29 d) showed a reduction of LV function measured by EF (> 20 points) and a marked increase in LV diastolic and systolic volumes (EDV and ESV) (Fig. 3B-D). Thus, a 90-min occlusion followed by 29 d of reperfusion caused an infarct large enough to produce ischemic cardiomyopathy in the immunodeficient rats. There was no significant difference among the group in all these parameters, indicating that, before treatment, the severity of post-MI LV remodeling and dysfunction were comparable among all groups (Fig. 3B-G).

At 34 days after treatment, vehicle-injected rats showed a modest increase in LV end-diastolic (EDV) (33.1 ± 64.5 µL) and systolic (ESV) (14.5 ± 56.1 µL) volumes (Supplementary Fig. 2), and a negligible change in EF (0.5 ± 11.4%)(Fig. 3H). These data suggest that LV remodeling continued to progress from the treatment (30 d) to the last echocardiographic measurement (64 d).

Next, we evaluated the effect of BM MSCs and WJ MSCs on LV remodeling and dysfunction. At 34 d after treatment, ESV and EDV in the vehicle (157.0 ± 43.0 µL and 312.9 ± 56.7 µL), BM MSCs (145.1 ± 52.0 µL and 309 ± 74.6 µL), and WJ MSCs (161.3 ± 52.4 µL and 344.1 ± 71.0 µL) groups did not differ significantly among the experimental groups (Fig. 3B and C). Furthermore, there were no significant differences in the changes in ESV and EDV between before (Pre-TX) and after (Post-Tx) treatment with BM MSCs, WJ MSCs, and vehicle (Supplementary Fig. 4). Similarly, at 34 d post treatment, there were only small, statistically nonsignificant differences in EF among vehicle (50.1 ± 7.7%), BM MSCs (53.8 ± 7.5%), and WJ MSCs (53.8 ± 8.1%) groups (Fig. 3G). Although the increase in EF from pre-treatment to post-treatment was numerically greater in the BM MSCs (5.0 ± 9.4%) and WJ MSCs (3.1 ± 8.8%) groups than in the vehicle group (0.5 ± 11.5%), these differences were not statistically significant (Fig. 3H). Together, these data indicate that, when measured with echocardiography, BM MSC and WJ MSC administration had no statistically significant effect on LV function and remodeling.

### Effect of WJ MSCs and BM MSCs on LV Function Measured with Hemodynamic Studies

**Fig. 4:**
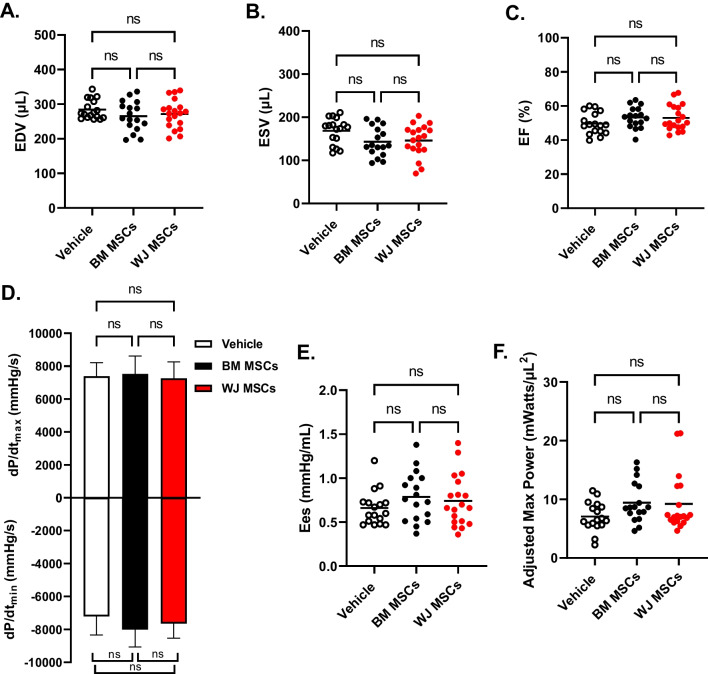
Hemodynamic assessment of LV function after BM and WJ MSC treatment. Conductance catheters were used to evaluate LV volumes [**(A)** end-diastolic volume (EDV) and **(B)** end-systolic volume (ESV)], **(C)** ejection fraction (EF), **(D)** LV dP/d*t*_max_ and LV dP/d*t*_min_, and load-independent indices of LV systolic function [**(E)** end-systolic elastance (Ees) and **(F)** preload adjusted maximal power]. Graphs with individual data points show mean as the horizontal line; the graph on panel D shows data as mean ± SEM for treatment groups consisting of vehicle (*n* = 17), BM MSCs (*n* = 17), and WJ MSCs (*n* = 19) groups; ns – not significant (two-way ANOVA)

Next, we performed an additional measurement of LV performance to evaluate the reparative efficacy of BM MSCs and WJ MSCs. Hemodynamic studies with conductance catheters were performed at 35 d after cell or vehicle injection (prior to euthanasia). The effects of WJ MSC and BM MSC administration on load-dependent (LV EF, LV dP/dt_max_, and LV dP/td_min_) and load-independent (end-systolic elastance (Ees) and preload adjusted maximal power) measurements of LV systolic function were assessed. There was no difference in EDV (vehicle: 284.2 ± 27.8 µL; BM MSCs: 265.4 ± 43.0 µL; WJ MSCs: 271.5 ± 41.5 µL) and ESV (vehicle: 168.5 ± 30.1 µL; BM MSCs: 143.2 ± 34.0 µL; WJ MSCs: 145.9 ± 37.0 µL) among the three groups (Fig. 4A and B). There was a modest, statistically non-significant improvement in EF in cell-treated groups compared with vehicle controls (vehicle: 49.7 ± 6.3%; BM MSCs: 53.3 ± 6.1%; WJ MSCs: 53.1 ± 7.5%) (Fig. 4C). The measurements of LV contractility with dP/dt_min_ and dP/dt_max_ also did not yield statistically significant differences (Fig. 4D). Lastly, load- independent measurements of LV function with end-systolic elastance and adjusted maximal power showed trends toward improvement in cell-treated groups relative to vehicle, but the differences did not reach statistical significance (Fig. 4E and F). Together, these data indicate that at the given dose, the injected cells did not produce statistically significant improvements in either load-dependent or load-independent parameters of LV function.

### Effect of WJ MSC and BM MSC Administration On Myocardial Fibrosis and Hypertrophy

**Fig. 5:**
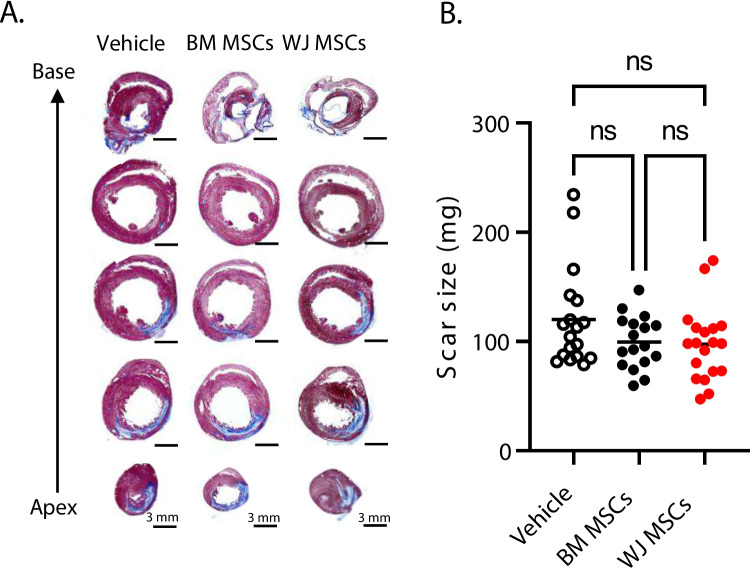
Histological assessment of scar size and fibrosis after BM MSC and WJ MSC administration with Masson’s trichrome staining. Each rat heart was dissected into five 3- mm tissue blocks (from apex to base). Each block was sectioned into 5-µm sections and subjected to Masson’s trichrome staining **(A)**. Planimetric measurements of the stained tissue sections were used to determine scar size **(B)**. All graphs contain individual data points with mean values in vehicle (*n* = 18), BM MSCs (*n* = 17), and WJ MSCs (*n* = 19) groups. Ns – not significant (two-way ANOVA)

**Fig. 6:**
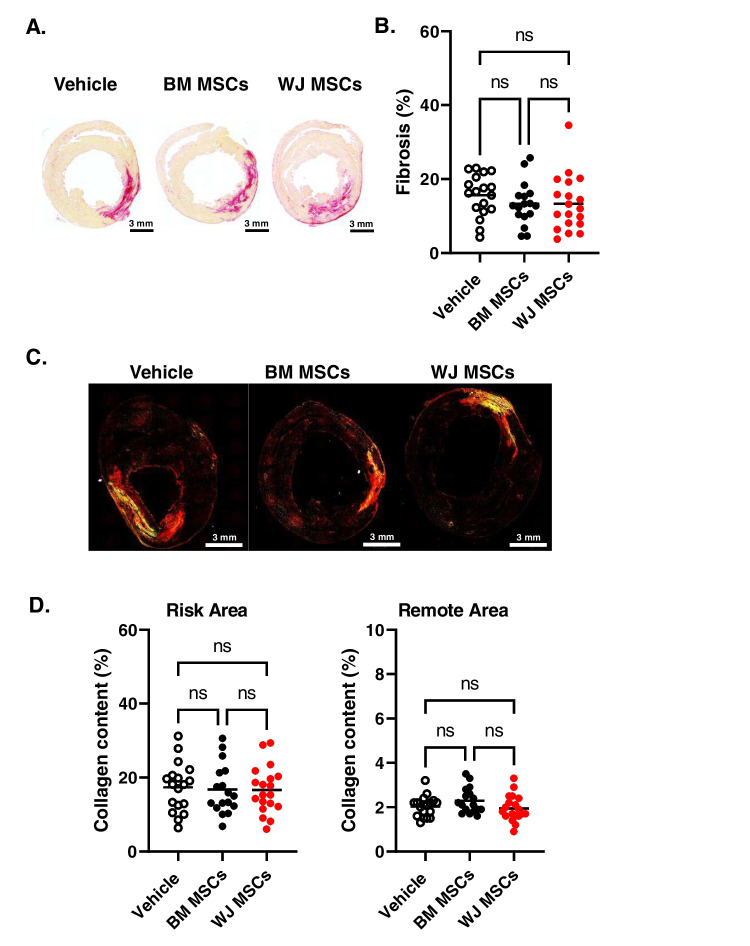
Histological assessment of scar size and collagen content after BM MSC and WJ MSC administration with picrosirius red staining. Mid-papillary heart sections were stained with picrosirius red **(A)**. Planimetric measurements of stained tissue sections were used to determine fibrosis **(B)**. All graphs contain individual data points with mean values invehicle (*n* = 18), BM MSCs (*n* = 17), and WJ MSCs (*n* = 19) groups; ns – not significant (two-way ANOVA)

**Fig. 7:**
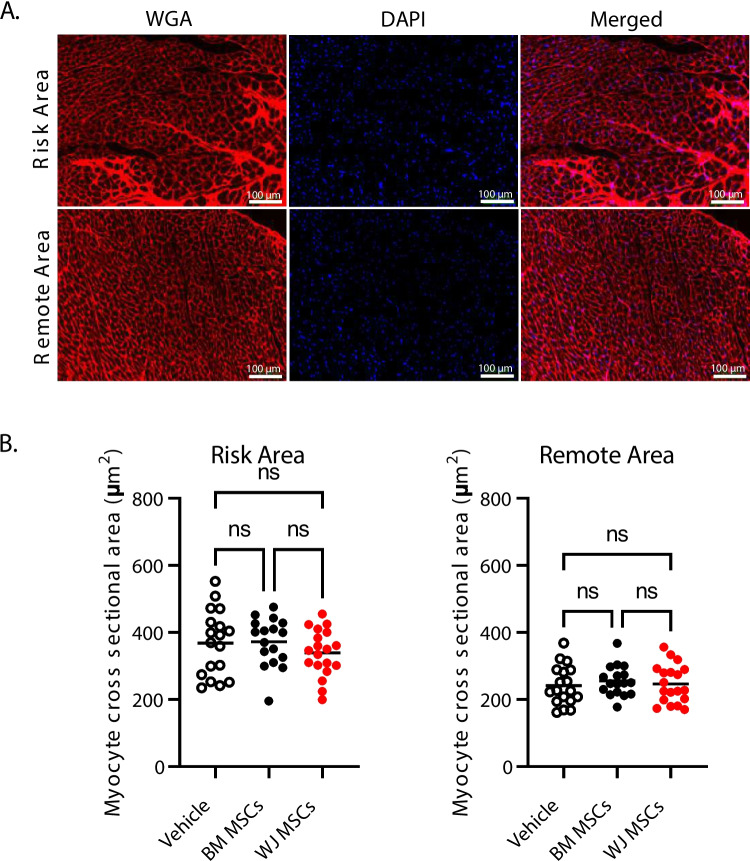
Histological assessment of cardiomyocyte hypertrophy after BM MSC and WJ MSC administration. Mid-papillary heart sections were stained with wheat germ agglutinin (WGA; cell membranes; red) and DAPI (nucleus; blue) and imaged **(A)**. Cardiomyocyte cross-sectional area was measured in periinfarct and remote areas **(B)**. All graphs contain individual data points with mean values in vehicle (*n* = 18), BM MSCs (*n* = 17), and WJ MSCs (*n* = 19) groups; ns – not significant (two-way ANOVA)

Previous studies by our group showed modest reduction in myocardial fibrosis after cardiac-derived cell product administration chronically after MI [27, 28]. Thus, we examined the effect of WJ MSC and BM MSC administration on scar size. Thirty-five days after vehicle or cell administration, histological analyses were performed to evaluate scar size and collagen deposition. Hearts were sectioned transversally into five 3-mm tissue blocks from the apex to base and weighted. Tissue sections from each tissue block were stained with Masson’s trichrome, imaged, and subjected to planimetric measurements of scar size (Fig. 5A). Total scar size (in mg) was calculated with a combination of gravimetric and planimetric analyses (Fig. 5B). We found that compared to vehicle-treated hearts (118.0 ± 45.1 mg), there was a modest reduction in scar size after BM MSC administration (98.2 ± 23.9 mg), but it did not reach statistical significance. Similarly, administration of WJ MSCs resulted in reduced scar size (97.4 ± 33.5 mg) without statistical significance (Fig. 5B). Next, mid-papillary LV sections were assessed for collagen content with picrosirius red staining. The stained sections were imaged with both bright field and polarized light. Analysis of collagen content with brightfield showed a small reduction in the cell-treated groups as compared with the vehicle-injected hearts (vehicle: 15.6 ± 5.7%; BM MSCs: 13.4 ± 5.8%; WJ MSCs: 13.4 ± 7.5%); however, again, the differences did not reach statistical significance (Fig. 6A). Next, we performed assessment of regional changes in collagen content in sections images with polarized light (Fig. 6B). Quantitative analysis of collagen content in the LV risk area (vehicle: 17.4 ± 6.7%; BM MSCs: 16.8 ± 6.7%; WJ MSCs: 16.7 ± 6.4%) and remote area (vehicle: 2.0 ± 0.5%; BM MSCs: 2.3 ± 0.6%; WJ MSCs: 2.0 ± 0.6%) showed no statistical difference (Fig. 6C). Similarly, we did not observe any significant differences in myocyte cross-sectional area in the peri-infarct area (vehicle: 368.6 ± 97.9 µm^2^; BM MSCs: 372.5 ± 71.6 µm^2^; WJ MSCs: 338.7 ± 69.6 µm^2^) and remote area of the left ventricle (vehicle: 241.7 ± 58.6 µm^2^; BM MSCs: 255.7 ± 45.2 µm^2^; WJ MSCs: 246.5 ± 56.8 µm^2^) (Fig. 7A and B). These data indicate that neither of the injected cell types affected cardiac fibrosis or hypertrophy.

### Effect of Cell Therapy on Peripheral Blood and Myocardial Tissue Immune Cell Count

**Fig. 8:**
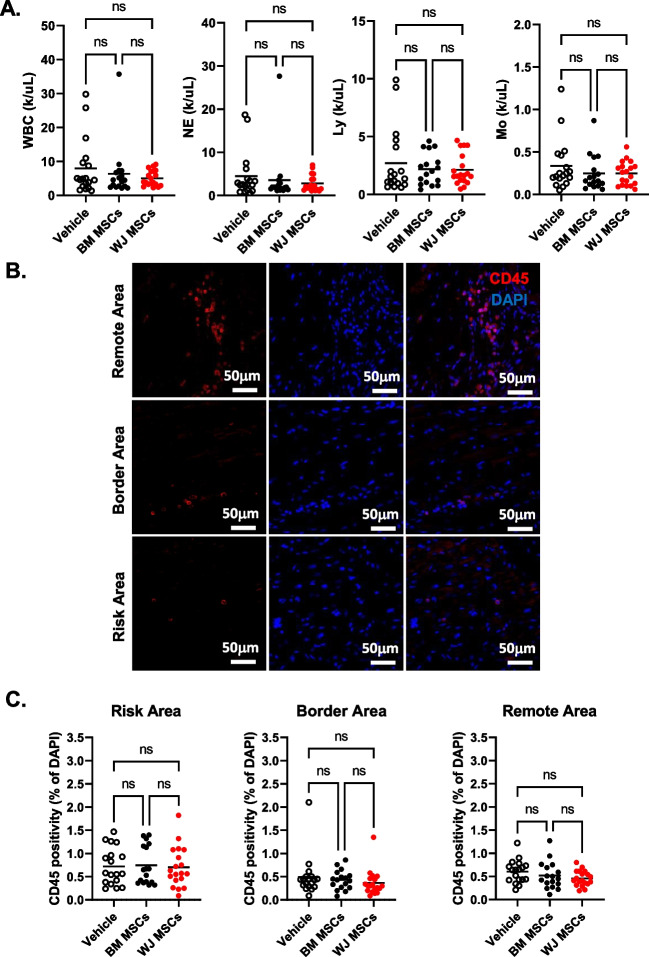
Effect of BM MSC and WJ MSC administration on circulating and myocardial tissue immune cell count. Peripheral blood white blood cells (WBC), neutrophils (NE), lymphocytes Ly), and monocytes (Mo) at sacrifice (65 d) were assessed with hemavet **(A)**. Representative images of mid-papillary heart sections stained with CD45 (red) and DAPI (blue) to identify tissue immune cells **(B)**. Quantitation of cardiac tissue CD45^Pos^ immune cells **(C)**. All graphs contain individual data points with mean values invehicle (*n* = 18), BM MSCs (*n* = 17), and WJ MSCs (*n* = 19) groups; ns – not significant (two-way ANOVA)

Peripheral blood cell counts including neutrophils, neutrophil to lymphocyte ratios, and neutrophils to platelets ratios are predictors of heart failure outcomes in patients [29–31]. We collected rat peripheral blood at 65 d after MI (during euthanasia) to perform a complete blood count. We found that BM MSCs and WJ MSCs had no effect on total white blood cell count, neutrophils, lymphocytes, and monocytes (Fig. 8A). These data indicate that intramyocardial BM MSC or WJ MSC injection is insufficient to affect peripheral blood immune cell counts.

Recent findings by us and others suggest that the injected cells improve LV function and fibrosis via immunomodulatory effects [11, 32]. Accordingly, we assessed local changes in the immune cell content in the heart at 65 d after vehicle, BM MSC, and WJ MSC administration. The immune cell content was evaluated by staining for the panhematopoietic marker CD45 (Fig. 8B). Enumeration of CD45 positive cells in the heart sections showed no effect of BM MSC and WJ MSC administration on immune cell content in the ischemic, border, or nonischemic regions (Fig. 8C). Together, these data suggest that intramyocardial injection of BM MSCs and WJ MSCs had no effect on myocardial inflammation in this immunocompromised model of chronic ischemic cardiomyopathy.

## Discussion

Despite two decades of investigation, fundamental problems regarding cell therapy for the treatment of heart failure remain [1]. These include identifying the best cell type, dose, route of administration, and timing of administration after MI. In addition, the mechanism(s) whereby injected cells modulate myocardial repair is not fully elucidated [1, 33]. Here, we tested the reparative potential of WJ MSCs and BM MSCs in a model of chronic ischemic cardiomyopathy induced with a reperfused MI. We found that neither cell type improved cardiac function and remodeling or diminished fibrosis. Other structural abnormalities such as cardiomyocyte hypertrophy and myocardial inflammation also failed to showed improvement after BM MSC and WJ MSC administration when compared with vehicle controls.

The main objective of our study was to test the efficacy of WJ MSCs in chronic ischemic cardiomyopathy. The rationale for using these cells is that they can be isolated from the umbilical cord, which is considered biological waste after delivery [17, 20, 21]. Thus, there is no need for invasive procedures to obtain tissue samples for cell isolation, like those necessary for isolation of cardiac cells (c-kit^Pos^ cells, cardiac mesenchymal cells, cardiosphere-derived cells) or BM-derived cells via aspiration (BM MSCs, BM EPCs). Furthermore, the abundance of umbilical cord tissue allows isolation of large quantities of cells, which obviates the need for extensive *in vitro* cell expansion to obtain suitable number for cells for treatment in human patients [17, 20, 21]. Because the umbilical cord is considered a neonatal tissue, WJ MSCs are more primitive than cells isolated from adult tissues including BM MSCs [14, 16, 34, 35]. When compared with BM MSCs, WJ MSCs show lower expression of markers of replicative senescence (β-galactosidase) and higher proliferative capacity with lower population doubling time [36, 37]. Although there is a possibility that the genetic background of the umbilical cord donor could affect the reparative potential of WJ MSCs, these cells are otherwise morbidity free, in contrast to other cells types utilized as a cell-based product, which are isolated from adult donors where other factors could influence the quality of the cell product, like age, comorbidities, medications, etc [17]. Additionally, similar to BM MSCs, WJ MSCs show low immunogenicity, which makes them suitable for off-the-shelf allogeneic treatment [15, 17, 20, 21, 35, 38]. Taken together, these considerations suggest that WJ MSCs may be a promising cell product for the treatments of cardiovascular disease.

Extensive studies in animal models of acute MI and in patients show that BM MSCs are safe and ameliorate LV fibrosis, structure, and function in patients with chronic heart failure [1, 2]. Less is known about the safety and therapeutic efficacy of WJ MSCs in patients with cardiovascular disease. A small study (*n* = 10), showed that intracoronary infusion of WJ MSCs at a dose of 3 × 10^7^ cells in patients 5–7 days after MI is safe. Administration of cell had no adverse effects measured by ECG signs of myocardial ischemia, coronary epicardial blood flow, and myocardial perfusion [39]; however, treatment efficacy was not studied. In a similar study, Gao [40] et al.enrolled 116 patients with acute-ST elevation MI, which were randomly assigned to receive placebo or intracoronary infusion of 6 × 10^6^ WJ MSCs at 5–7 days after reperfusion therapy. There were no adverse effects up to 18 months after cell infusion. At the 4-month follow-up, treated patients showed an increase in myocardial viability evaluated with PET and improved perfusion within the infarcted region. Furthermore, at the 18-month follow-up, treated patients showed improvement in LVEF and reduction in end-systolic and diastolic volumes compared with placebo-treated patients [40]. Lopez et al.reported that rats receiving 3–10 × 10^6^ syngeneic WJ MSCs intravenously at 24–48 h after permanent coronary ligation had an improvement in EF at 25–31 weeks after injection [41]. Together, this initial evidence suggests that WJ MSCs may promote myocardial repair when injected intramyocardially or intravenously acutely after the onset of MI; however, more rigorous studies are needed to confirm this observation. Whether the same reparative potential can be observed when WJ MSCs are injected chronically after MI is unknown.

In the current study, we tested the therapeutic efficacy of BM MSCs and WJ MSCs in a chronic model of reperfused ischemic cardiomyopathy in rats. We have used this model in previous studies to test the therapeutic efficacy of c-kit^Pos^ cardiac cells and cardiac mesenchymal cells (CMCs) delivered by various routes (intramyocardial, intracoronary, intravenous) using Fisher immunosufficient rats and immunodeficient nude rats [10, 27, 28, 42–46]. We found that in this model, cell therapy improved LV structure and function with single or multiple injections [10, 27, 28, 42–46]. Thus, we are confident that our model is suitable to test the therapeutic potential of new cell types, such as WJ MSCs. In the present study, we used BM MSCs with the expectations that WJ MSCs would show superior therapeutic efficacy. Although the lack of improvement in LV structure and function is a new finding, the finding that BM MSCs showed no effects on myocardial repair is consistent with some previous studies. There are reports by others demonstrating that the efficacy of BM MSCs in chronic ischemic cardiomyopathy is cell donor-dependent. Befhar et al [47] tested the efficacy of BM MSCs from 11 donors in a mouse model of chronic heart failure. Out of 11 BM MSC cell lines, only three improved LV structure and function when injected intramyocardially in the chronic phase after MI. Further analysis showed that expression of cardiogenic factors was a strong predictor of the reparative properties of BM MSCs [47]. The CONCERT-HF trial in patients with heart failure showed that autologous BM MSCs, delivered via the transendocardial route, produced an improvement in quality of life measured with the Minnesota Living with Heart Failure Questionnaire score, but no improvements in major adverse cardiac events, LV EF, LV volumes, scar size, 6-min walking distance, and peak oxygen consumption [48]. Other clinical trials, however, have found evidence of efficacy with BM MSCs in chronic heart failure [1–4]. The reparative potential of BM MSCs in patients with chronic ischemic cardiomyopathy remains unclear.

Although the results of our study agree with some published studies regarding the therapeutic potential of BM MSCs for the treatment of chronic heart failure, the utility of WJ MSCs remains underexplored. Our results revealed that intramyocardial injection of WJ MSCs (3 × 10^6^ cells) in the chronic phase of a reperfused MI in nude rats did not improve LV structure and function. To our knowledge, no previous study has examined the reparative potential of WJ MSCs in chronic MI-induced ischemic cardiomyopathy. Nevertheless, the umbilical cord is a source of other cell populations. The most common is described as umbilical cord MSCs (UC MSCs). Although WJ MSCs and UC MSC are isolated from the same tissue, there are some minor differences. UC MSCs are isolated as a mixture of Wharton’s jelly MSCs and perivascular cells including endothelial and smooth muscle cells and pericytes, while WJ MSCs are isolated from the umbilical cord after removing vein and arteries. The fact that UC MSCs are more heterogeneous compared with WJ MSCs could affect their reparative potential. Liu [49] et al. showed that UC MSCs improved LV function in a pig model of chronic heart failure induced with ameroid constrictor implant. In their study, UC MSCs were injected intracoronarily at 4 weeks after MI and intravenously at 5 and 6 weeks after MI. Measurements of LV function at 8 weeks after MI showed improvement in EF and reduction in ESV in the cell-treated group compared with vehicle. These data suggest that multiple doses of UC MSCs provide a small but significant improvement in LV function. This small study (*n* = 6) did not test durability of the treatment, as the final echo was performed two weeks after the last dose of cells [49]. Bartolucci et al. [50] conducted a phase 1/2, randomized, controlled clinical trial in patients with heart failure. In this small study (*n* = 15/group), 1 × 10^6^ allogeneic UC MSCs/kg or placebo were injected intravenously in patients with chronic heart failure. Cell-treated patients had no adverse events related to cell infusion and none of the patients developed alloantibodies against UC MSCs, suggesting that the treatment is safe. Cell-treated patients exhibited a significant improvement in LV EF tested at 3, 6, and 12 months with echocardiography and MRI. In addition, cell-treated patients showed an improvement in New York Heart Association functional class and quality of life assessed with the Minnesota Living with Heart Failure Questionnaire [50]. Together, these studies suggest that UC MSCs are effective in improving LV function when injected in the chronic phase after MI. The studies discussed above raise the question as to whether there is a functional difference between a pure population of WJ MSCs and mixed UC MSCs in their reparative potential, particularly when injected in the chronic phase of MI. These could be explored in the future studies.

In our studies, we utilized immunodeficient nude rats that lack T cells. This is a potential limitation of the study. There is a growing evidence that T cells have adverse effect on ventricular remodeling in chronic heart failure [51–55]. Studies in mice revealed that T cells are recruited to failing hearts to activate fibroblasts and contribute to excessive fibrosis and progressive failure of pump function [51–55]. Furthermore, it has been reported that BM MSCs can antagonize T cell proliferation and activation both in vitro and in vivo [1]. Some recent reports indicate that WJ MSCs may have an inhibitory effects on T cells similar to the effect of BM MSCs [56]. Thus, future studies would have to test reparative potential of WJ MSCs in immunesufficient animal models of heart failure.

In conclusion, our results demonstrate that human BM MSCs and WJ MSCs have limited reparative potential when injected in the chronic phase of MI in immunodeficient rats. Future studies may focus on delineating reparative differences in other cell types isolated from the umbilical cord that are included in UC MSC preparations.

### Supplementary Information

Below is the link to the electronic supplementary material.

Supplementary file1 (PDF 576 kb)

## Data Availability

Data will be provided by the authors unpon reasonable request.
