# Supplementary material for: Bone Marrow and Wharton’s Jelly Mesenchymal Stromal Cells are Ineffective for Myocardial Repair in an Immunodeficient Rat Model of Chronic Ischemic Cardiomyopathy
Source: Stem Cell Rev Rep. 2023 Jul 28;19(7):2429–46. doi: 10.1007/s12015-023-10590-6 (PMC10579184; doi:10.1007/s12015-023-10590-6)
Supplement: Supplementary file 1 — Supplementary file1 (PDF 576 kb) [file 12015_2023_10590_MOESM1_ESM.pdf]

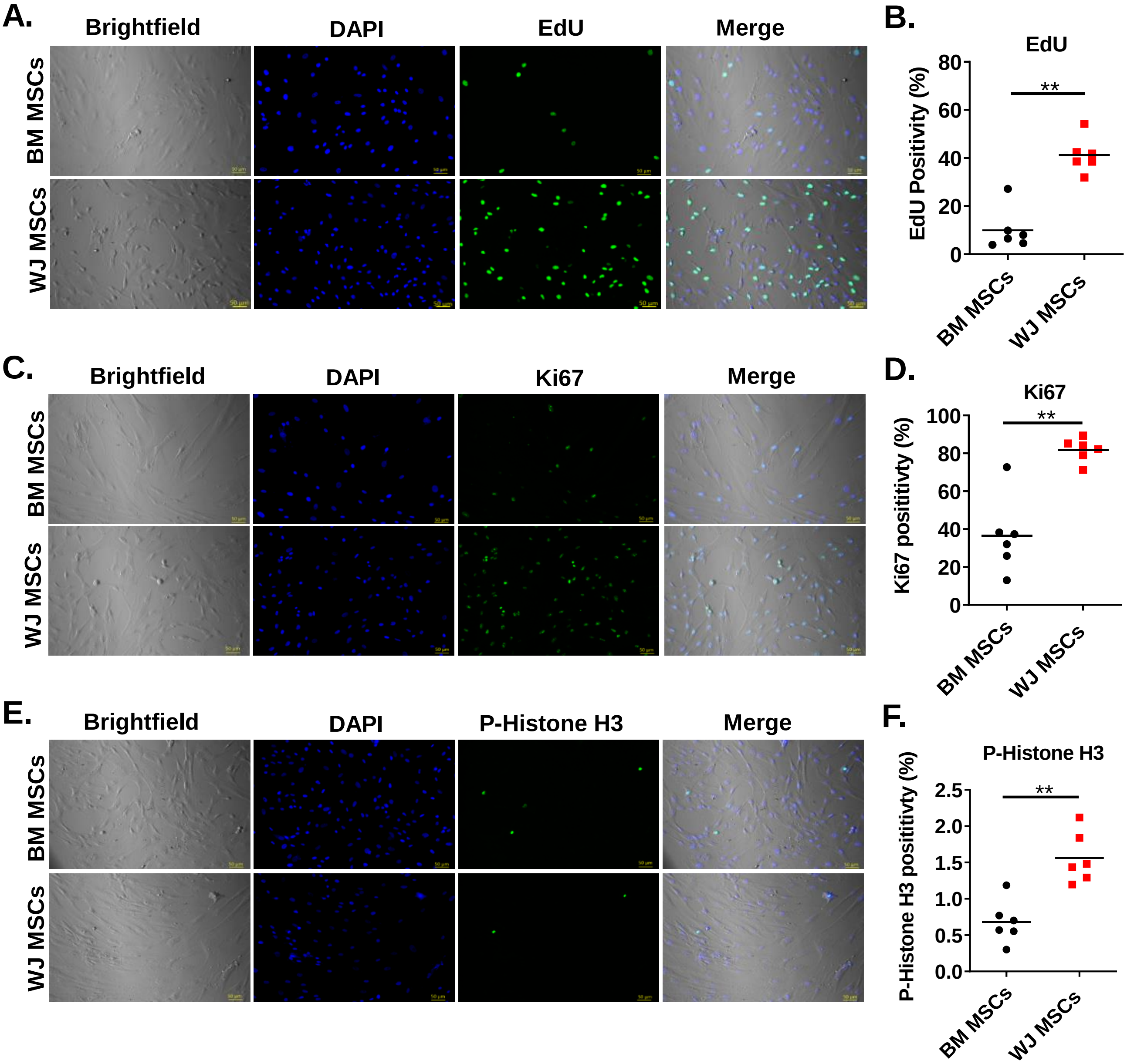

Supplementary Figure 1. Proliferative capacity of BM and WJ MSCs. Synchronized cultures of BM MSCs and WJ MSCs were imaged to detect EdU incorporation **(A)**, Ki67 **(C)**, and phosphor-histone H3 expression **(E)**, and quantified **(B, D, F).** Data are shown as individual data points, horizontal lines are mean values. BM MSC n=6, WJ MSCs n=6; **P<0.01 (unpaired t test).

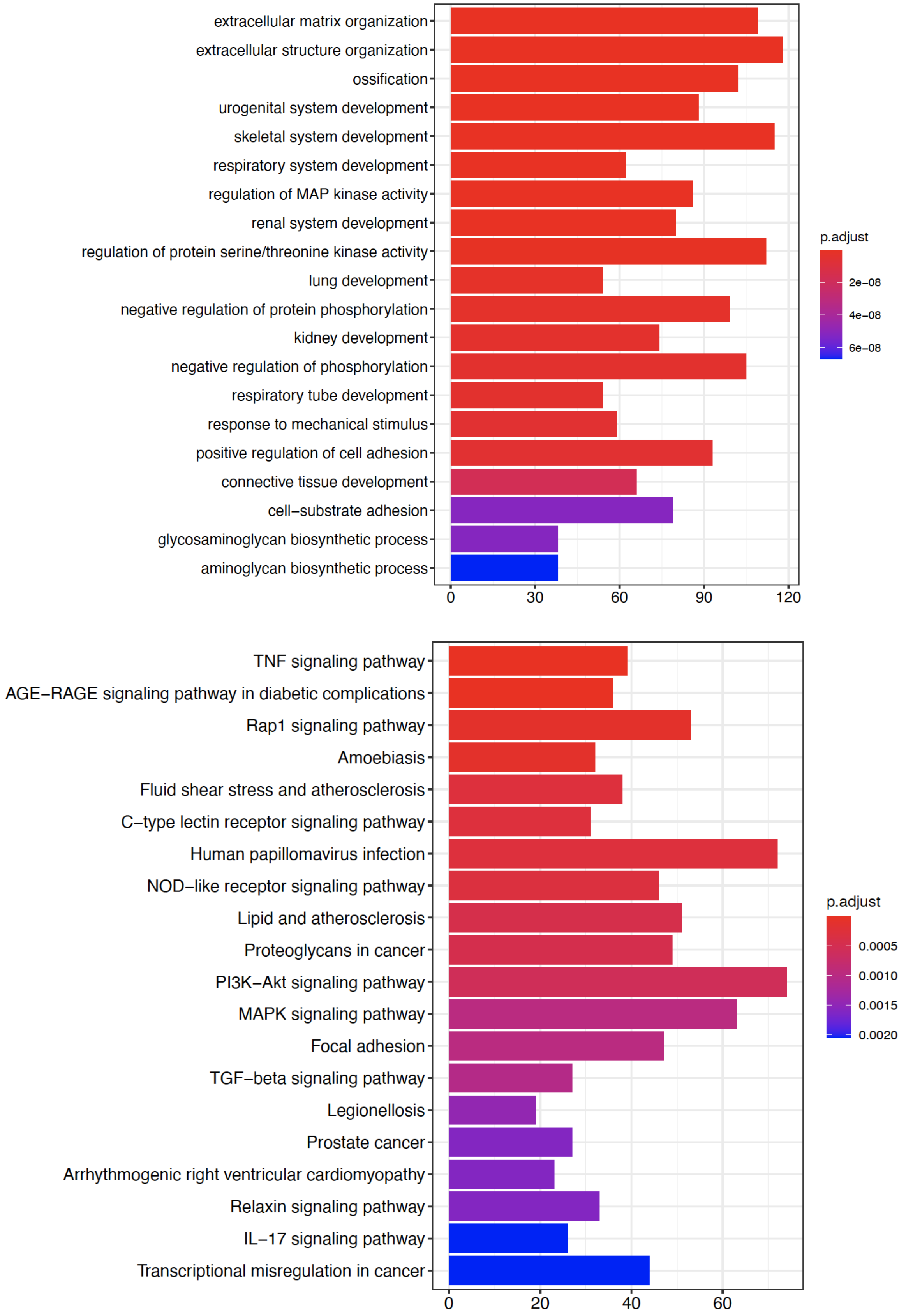

Supplementary Figure 2. The top 20 enriched GO biological process (A) and KEGG pathways (B) for WJ MSCs vs BM MSCs.

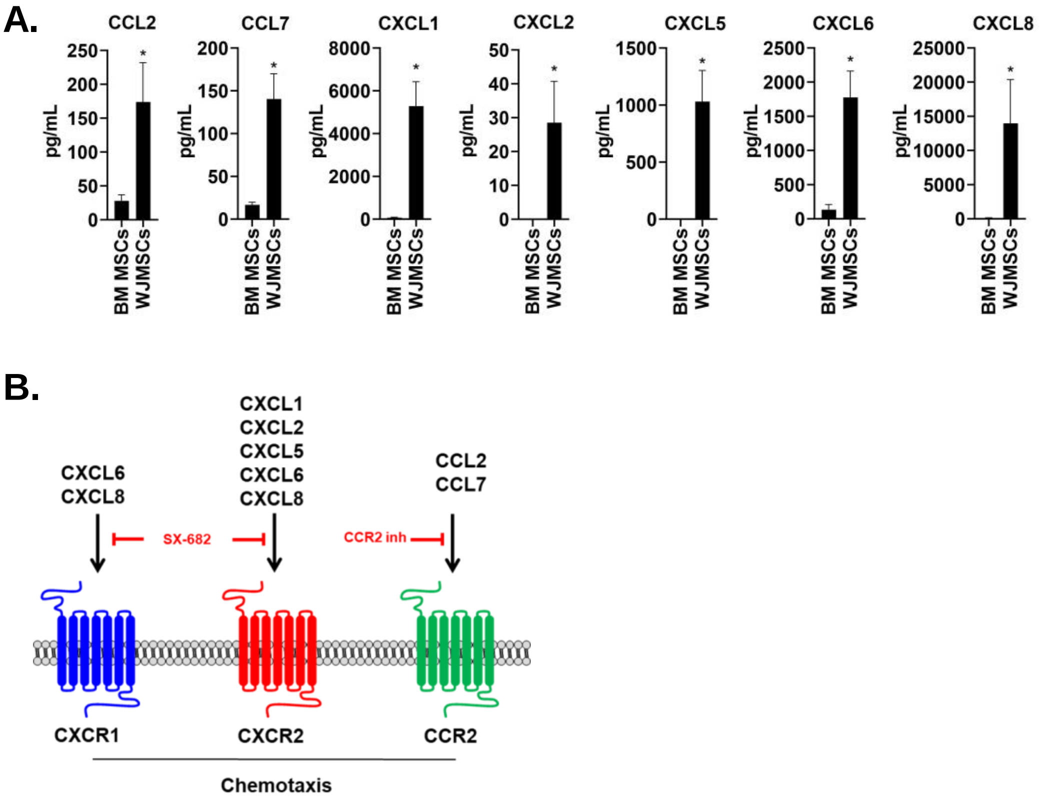

Supplementary Figure 3. BM and WJ MSC secrete CC and CXC chemokines. Cytokine array of conditioned media collected from WJ MSC and BM MSC (A). BM MSCs and WJ MSCs secrete chemokines targeting CXCR1, CXCR2, and CCR2 (B).

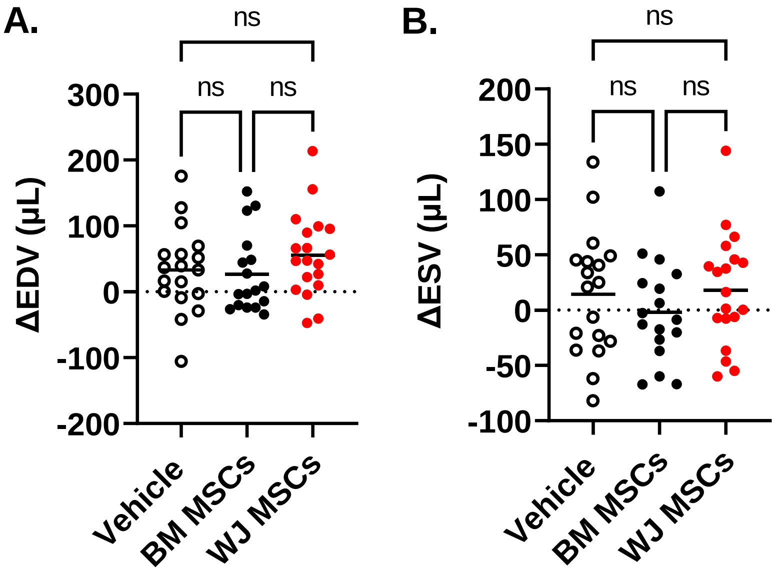

Supplementary Figure 4. Impact of BM and WJ MSC treatment on ventricular volumes. Rats were subjected to serial echocardiogram collection corresponding to pre-treatment (Pre-Tx), and post-treatment (Post-Tx) with cells (WJ MSC or BM MSCs) or vehicle. Change between an associated time points, ventricular volumes (**A** end-diastolic volume [EDV] and **B** end-systolic volume [ESV])) were calculated for each group. Graphs contain individual data points with mean values for treatment groups consisting of vehicle (n=18), BM MSCs (n=17), and WJ MSCs (n=19); ns – not significant (two-way ANOVA).
